# Classification of the oesophageal perforation

**DOI:** 10.1186/s40001-024-01910-8

**Published:** 2024-07-01

**Authors:** Friederike Harrich, Wolfram Trudo Knoefel, Edwin Bölke, Matthias Schauer

**Affiliations:** Universitätsklinikum Düsseldorf, Moorenstr. 5, 40225 Düsseldorf, Germany

**Keywords:** Esophagus, Perforation, Classification, Esophageal surgery

## Abstract

**Objectives:**

Esophageal perforations are a complex clinical scenario that have been poorly studied. To date, there is no grading of esophageal perforations, the reason being that the outcome is very heterogeneous, because the perforation is very heterogeneous. A grading of the severity of the perforation may guide treatment, and could ultimately affect morbidity and mortality.

**Methods:**

The observation period of the study was four years. All patients with a perforation of the esophagus aged 18 to 90 years were included. All anastomotic insufficiencies or fistulas after surgery of the esophagus were excluded. The cause of the injury and the time interval between the event and the start of therapy were analyzed. The severity of each perforation was classified based on the results of a diagnostic CT scan, gastroscopy as well as clinical and laboratory findings. Therapy and signs of infection were evaluated. Endpoints of the study were patient recovery or death. The study was conducted as a retrospective single-center study at a university hospital of Düsseldorf. The study has been approved by the review board. Patients gave their informed consent before data collection. All data were analyzed using SPSS 29 (IBM SPSS Statistics software).

**Results:**

Age, gender and cause of the esophageal perforation did not impact significantly on overall survival. The duration of injury > 24 h (*p* = 0.01), presence of mediastinitis (*p* = 0.01) and necrosis of the esophagus (*p* = 0.02) were associated with an unfavorable outcome. The correlation of the clinical grading of the severity of the perforation based on the endoscopic, radiological and clinical findings with the overall survival of patients was significant. Patients categorized into the four grades of severity (I–IV) had an overall survival of 100%, 100%, 70% and 50%, respectively.

**Conclusion:**

The severity of esophageal perforations can be systematically rated grades I to IV based on the radiological, endoscopic and clinical findings at diagnosis. Due to the grading and its correlation to the overall survival, a comparison of patients, their treatment and outcome becomes possible. In future, the grade of a perforation may guide treatment, and therefore affect morbidity and mortality.

## Introduction

Esophageal perforations are a highly complex clinical scenario and can present a life-threatening emergency situation. To date the inhomogeneity of the severity of the perforation, and the lack of a systematic grading of the perforation have made it impossible to predict the lethality of a given perforation. Moreover, studies investigating patients with esophageal perforation are not comparable and show a great variety of results and difference in outcome [5, 6, 12, 24, 27].

The literature on this topic is characterized by inconsistent patient collectives, studies with only a small numbers of cases and single case reports. It discusses anastomotic insufficiencies together with all other esophageal perforations and does not differentiate between different types of perforations making a standardization difficult [3, 5, 10, 11, 17, 27].

The cause of esophageal injuries is manifold it includes swallowed foreign bodies, Boerhaave’s syndrome or iatrogenic injuries [27–29]8. The risk of a perforation during endoscopy is 0.03%. It may increase to 17% during a therapeutic intervention related to the diagnosis or treatment of the underlying disease. In fact about half of all esophageal perforations occur during endoscopic examinations and procedures [1, 4, 6, 9, 11, 22].

Independent of its cause, the injury needs to be quickly diagnosed and adequately treated.

Today, depending on the appearance of the perforation a broad range of therapeutic options can be applied. More than 50% of esophageal perforations are treated endoscopically [8, 19, 21].

In this study, our patients with an esophageal perforation were categorized according to the severity of their injury which was rated based on a CT scan, endoscopy as well as their clinical and laboratory findings. All patients had esophageal perforations without a previous esophagectomy.

The aim of this study was to evaluate, if a reproducible, easy to apply grading of esophageal perforations and its correlation to the clinical outcome is possible.

For this a classification of esophageal perforations must precede the evaluation of potential treatment options. It is believed that this classification may create a comparability of clinical outcomes in the future.

## Patients and methods

The observation period of this retrospective study was 4 years. All patients with an esophageal perforation treated in our hospital were included. They amounted to 38 patients. Cases with a primary surgical procedure of the esophagus, e.g., an esophagectomy with an anastomotic insufficiency were excluded. The study was review board approved (EK HHU: 4664). Patient consents were obtained. Patient data were anonymized and subsequently analyzed. The raw data are available to the reader on request to the author. All data were analyzed using SPSS 29 (IBM SPSS Statistics software). The study was conducted as a single-center study in a maximum care hospital.

The cause of injury, the findings on the CT scan, endoscopic findings and the extent of perforation were analyzed, as well as the time interval between the event and the start of therapy. Therapy and clinical signs of infection were evaluated thereafter.

Endpoints of the study were defined as healing of the esophageal perforation or death of the patient in the further clinical course.

Statistics: Comparison of numeric data was performed with Student’s *t*-test. Comparison of categoric data was performed with Fisher’s exact test. The threshold of statistical significance was set at 0.05. Statistical analysis was performed with SPSS 29 for windows (SPSS, Chicago, Illinois, USA).

## Results

The male-to-female ratio in the study was 58% (m): 42% (f). The median age of study participants at the time of perforation was 63, and the range of age was 27–87 years.

38% of the patients in the study were transfers from other hospitals.

**Fig. 1:**
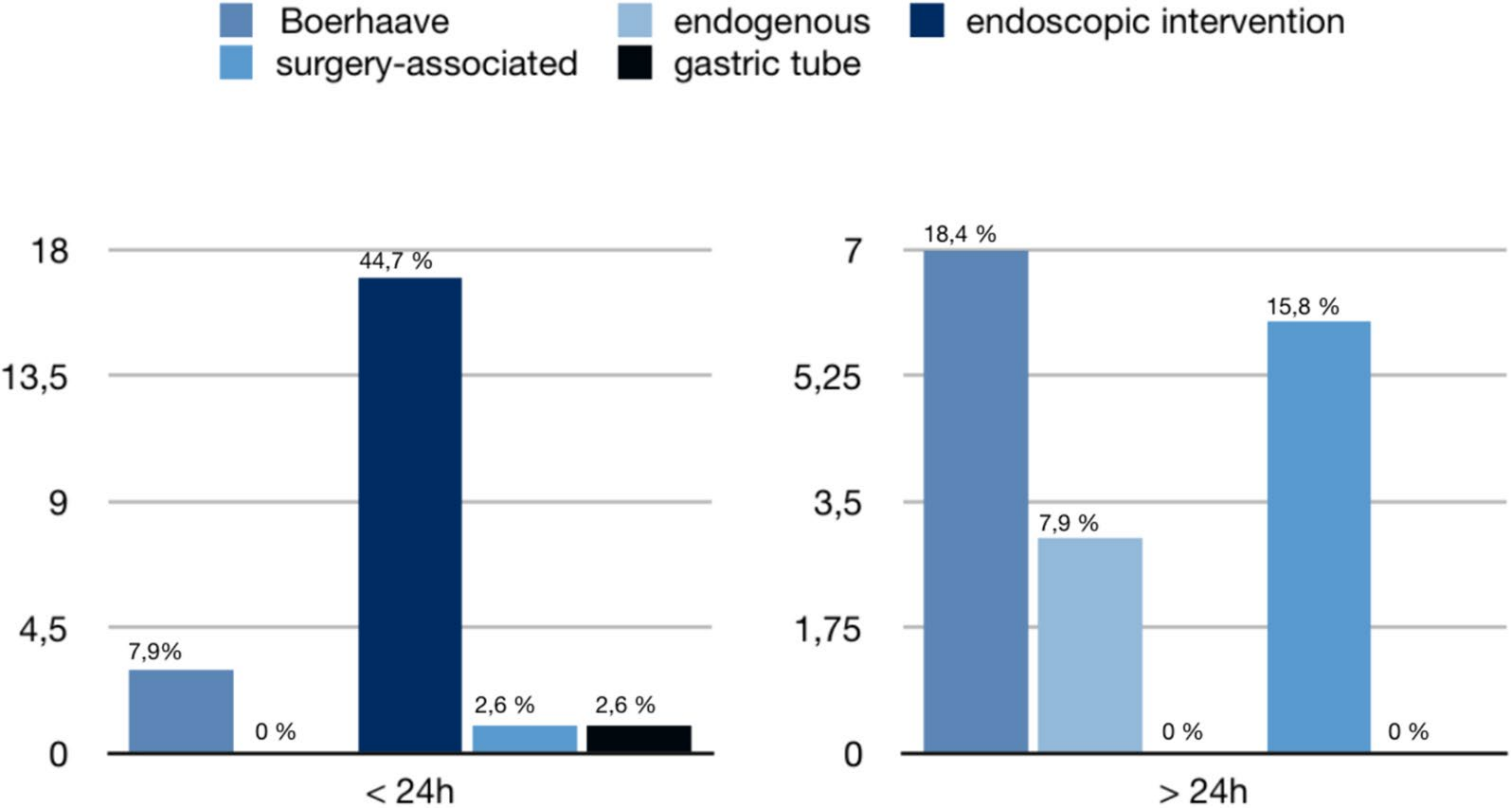
Hours between perforation and hospitalization < 24 h and > 24 h. Significant correlation between duration of injury > 24 h (*n* = 17) and < 24 h (*n* = 21) with survival (*p* = 0.02) on Fisher’s exact test

Age, gender and cause of the perforation did not significantly correlate to the survival rate (*p* > 0.05). The correlation of the duration of injury < 24 h (*p* = 0.01), presence of a mediastinitis (*p* = 0.01) or necrosis of the esophagus (*p* = 0.02) and degree of injury (*p* = 0.02) with survival were significant in the Fisher’s exact test (Fig. 1).

Table 1 shows the causes of esophageal perforation in percent. It is notable that 45% of all esophageal perforations occur after endoscopy [transesophageal echocardiography (TEE, 5%), endoscopic retrograde cholangiopancreaticography (ERCP, 5%), esophagogastroduodenoscopy (EGD, 16%), balloon dilatation (16%)]. Surgically induced were perforations due to gastric banding (3%), fundoplication (6%), goiter surgery (3%) and surgery of the cervical spine (6%). The other iatrogenic causes were PEG tube removal (2%) with esophageal injury. A spontaneous injury occurred in terms of esophageal cancer or chemotherapy. Other perforations were induced by swallowed foreign bodies.

**Table 1:** Causes of esophageal perforation

Cause	Percentage (%)
Endoscopies	45
Surgically induced perforation	18.4
Other iatrogenic cause, pooled	2
Spontaneous	2.5
Boerhaave	26.3
Other	5.8

Surgical therapy was performed in most cases (71%), while less than 30% were treated endoscopically (Table 2). This was performed with, e.g., nasal tube, stent or endovacuum. Irrespective of the kind of treatment 21% of all study participants with an esophageal perforation died during their hospital stay. Of the patients undergoing surgery 26% died within 90 days post-surgery, 74% survived post-surgery. There was no positive correlation between the surgical treatment of the perforation and patient outcome.

**Table 2:** Conservative treatment vs. surgical treatment of patients included in the study

	Percentage (%)
Surgical treatment (71%)	
Esophagectomy	46
Overstitching and plastic reconstruction	43
Small bowel interposition	11
Conservative treatment (29%)	
Gastric tube	29
Intestinal stent	53
Endovac therapy	18

Figure 1 shows how quickly patients were treated after perforation. Figure 1 on the left shows the percentage of patients hospitalized within less than 24 h after perforation, and Fig. 1 on the right shows the percentage of patients hospitalized after more than 24 h. Most patients with an iatrogenic perforation following an endoscopic intervention were diagnosed and treated right away (45%), whereas patients with Boerhaave’s disease were often diagnosed time-delayed (18%). Endogenous perforations were due to esophageal carcinoma. Surgical associated perforations occurred after surgery of the goiter or the cervical spine.

Table 2 shows the percentages of the individual therapies. The options for the different therapies were in 71% of cases surgical (46% esophagectomy, 43% primary suture with plastic reconstruction or 11% small bowel interposition) and in 29% of cases endoscopically (29% gastric tube, 53% intestinal stent and 18% endovacuum therapy).

An aspect of the evaluation was the very heterogeneous clinical, endoscopic and radiologic appearance of the disease, which is why we investigated the following grading for the prediction of lethality. A systematic grading of all perforations (grades I to IV) was performed. Table 4 shows Grade I was defined as a covered perforation with air in the mediastinum detected on a CT scan without clinical evidence of mediastinitis or sepsis or an endoscopic finding. Grade II shows a fresh perforation with leakage of oral contrast medium or fluid mediastinal retention on CT scan without clinical evidence of mediastinitis or sepsis. Grade III describes an esophageal perforation on CT scan, an endoscopic view outside the esophagus and presence of mediastinitis or sepsis. Grade IV presents an older perforation with a persistent fistula, long-stretch rupture or necrosis proved on CT scan and endoscopy, clinically mediastinitis and sepsis. Grade I and II perforations can only be seen on a CT scan. Laboratory findings, clinical findings and endoscopy are unsuspicious. By comparison grade III and IV perforations can be detected on a CT scan, endoscopically and clinically. Consequently for all patients CT scans, endoscopy results, clinical presentation and laboratory findings were taken into account for the grading and subsequent categorization of the perforations.

Reflecting the different clinical courses of the patients, we identified a significant correlation between the grade of the perforation and the clinical outcome and the associated lethality (*p* < 0.001). Table 3 shows that esophageal perforations type I and II have a survival rate of 100%, while grade III and IV have significantly worse outcomes with a lethality of 85% and 50%, respectively. All patients with an unfavorable outcome in groups III and IV died due to a prolonged sepsis with a multiorgan failure. Most patients with grade I and II perforations had a conservative therapy in 100% and 62.5%, respectively, while patients with grade III and IV perforations had a surgical therapy in 92.3% and 100%, respectively. Patients undergoing an esophagectomy as an emergency procedure (31.5% of all patients) had a two-step operation with a gastric tube reconstruction after convalescence. The additional surgical trauma and therefore the postoperative morbidity and mortality especially with regard to an anastomotic insufficiency after reconstruction was minimized. In the emergency situation the esophagectomy was standardized as a thoracotomy with removal of the esophagus, a cervical esophagostomy, blind closure of the stomach, a pleural lavage and insertion of two 24 Charriere chest tubes. Reconstruction was performed after convalescence.

**Table 3:** Lethality depending on the level and complexity of perforation

Type		Patients (%)	Surgical therapy (%)	Conservative therapy (%)	Survival (%)
I	Covered perforation with air in the mediastinum, no evidence of mediastinitis or sepsis	13.2	0	100	**100**
II	Fresh perforation with radiological leakage or retention without evidence of mediastinitis or sepsis	21	37.5	62.5	**100**
III	Cervical, thoracic or cardia perforation or mediastinitis or pleural empyema or peritonitis	34.2	92.3	7.7	**85**
IV	Older perforations with persistent fistula, mediastinitis, possibly esophageal necrosis, possibly long-stretch rupture or possibly sepsis	31.5	100	0	**50**

The percentages in the columns “Surgical therapy” and “Conservative therapy” refer to the absolute number of patients in each type

The correlation between grade and survival is significant (*p* < 0.001)

One patient with a grade II perforation was recategorized after 3 days into a grade III with a more extensive rupture with an endoscopic view into the mediastinum while changing the endovac. This patient was undergoing a primary suture as a thoracoscopic procedure with a muscle flap and an endoluminal vacuum therapy, pleural lavage and two 24 Charriere chest tubes. The esophagus healed in the further course and the patient survived.

In summary, duration of the injury before treatment and grading of the perforation as described above have a significant correlation to survival after esophageal perforation with a *p*-value of 0.02 and 0.01, respectively.

## Discussion

There is an overall high complication rate after surgical and after conservative therapy of an esophageal perforation [6, 22, 24–27, 30]. A correlation between an inhomogeneity of the severity of the perforation, therapies and the complication rate can be assumed.

Most studies show case reports or small cohorts of about 10 patients at most, the reason being the rare occurrence of esophageal perforation. One study described esophageal perforations of 29 patients, and there is a meta-analysis of 75 studies. A comparison of the patients of these studies proves difficult because without a grading of the esophageal perforation the comparability of the applied treatment options and their outcome is not rational. Heterogeneous survival rates are inevitable [2, 24].

Our patient collective was obtained from a German university hospital as a single-center study. It included 38% of patients transferred from other hospitals. This will definitely influence the duration of injury and the severity of the esophageal perforations as only grade III and IV perforations were transferred from other hospitals to our hospital for further surgical treatment. Therefore the number of high grade perforations, the relatively high amount of surgical treatments and the overall unfavorable survival rate of 78% are not representative for esophageal perforations in general. They do however reflect the situation of more severe patients. Nonetheless, this unique group of patients shows that a grading of the severity is possible, reasonable and can be applied to predict the mortality of a patient with a perforation and maybe direct a treatment decision.

The different manifestations of esophageal perforations can be classified into severity grades by listing the concomitant symptoms, the findings on CT scans with oral contrast medium and endoscopy. There is clearly a significant correlation between the severity of a perforation and lethality. The higher the degree of an esophageal perforation, the lower the patient’s probability of survival. By introducing different rating grades, all causes of perforation, the share of surgical patients and their survival become comparable in each single group. Of course, the applicability of the grading system and the comparability has to be validated before it can be transferred into clinical practice.

The increased homogeneity of the patient collective of this study may allow transferability of the results to other cases and clinical application of the presented grading of esophageal perforation, e.g., for further investigations concerning lethality of esophageal perforations and treatment options.

The general conclusion drawn in literature for or against conservative or surgical therapy was surgeon- and case-dependent [30]. Therapeutic endoscopic and conservative options are gastric tube, intestinal stenting and endovacuum therapy in combination with systemic antibiotics [21, 30]. Surgical options are suturing of the perforation, a plastic cover, e.g., with pleura, pericardium, gastric fundus, omentum majus or covering with a pedicled muscle flap or esophagectomy with a two-stage reconstruction [24, 26]. Our hospital treats grade I and II injuries mostly endoscopically and with a very good outcome. In patients with grade III and IV injuries surgical treatment has to be considered. 71% of all cases and even 96% of patients with grade III and IV perforations underwent a surgical procedure. 74% of all surgical patients survived. As it could often be shown in different studies, esophageal surgery is possible with a low morbidity and low mortality in high-volume centers [28, 29]. Regarding our patients, all of whom suffered already preoperatively from the perforation with clinical signs of mediastinitis and sepsis, we are convinced that the unfavorable outcome is caused by the underlying disease, not by the surgical procedure. As it can be derived from the criteria for a grade IV perforation which reflects patients with an esophageal necrosis, extensive esophageal ruptures with mediastinitis, pleura empyema and sepsis, all these patients that underwent esophagectomy were already preoperatively in bad general condition. A survival rate of 50% in these patients corresponds to the international literature [29]. Moreover, the correlation between the surgical treatment and the survival of our patients was not significant.

The review of the previous literature shows a very heterogeneous distribution of therapeutic strategies due to the lack of both a grading of the perforation and a standardized process to form the therapeutic decision [3, 4, 7, 9–11, 12, 13, 15–18, 20, 21, 25, 30].

Currently, the decision for or against a surgical treatment is made based on physician experience on a case-by-case basis. With the new grading of esophageal perforation presented in Table 4 it may soon become a more structured, rational decision. The grading may also allow a comparison of different patients collectives with different degrees of their perforation and eventually lead to a therapy recommendation.

**Table 4:** Classification and therapy in our patient collective

Type	Endoscopic-radiological	Therapy
I	Covered perforation with air in the mediastinum, no evidence of mediastinitis or sepsis	Conservative therapy, gastric tube, if necessary control with gastrografin swallow after 8 h
II	Fresh perforation with radiological leakage or retention without evidence of mediastinitis or sepsis	Intestinal stent, endovac if necessary, antibiotic therapy, control with gastrografin swallow after 8 h
III	Perforation with radiological leakage and endoscopic view into the mediastinum or pleura, possibly mediastinitis or pleural empyema	Surgical reconstruction with suturing, muscle or pericardial flap, internal and external drainage, clearing of an empyema, antibiotic therapy, endovac
IV	Older perforations with persistent fistula, mediastinitis, possibly esophageal necrosis, possibly long-stretch rupture and sepsis	Esophagectomy, two-stage reconnection, internal and external drainage, antibiotic therapy

### Summary of the proposed grading

#### Type I

The esophageal perforation is covered. There is air in the mediastinum, but no clinical evidence of mediastinitis or sepsis. In our patient collective, we indicated a conservative approach. A gastric tube was inserted immediately after diagnosis. The inflammatory parameters have always been checked regularly during the course of the procedure, and a gastrografin swallow was performed eight hours after diagnosis in order to detect a leakage.

#### Type II

The perforation is fresh with radiologically confirmed leakage or mediastinal retention. There is no evidence of mediastinitis or sepsis at this time. Grade II perforations were treated with an intestinal stent; endovacuum therapy was sometimes necessary. Furthermore, a calculated antibiotic therapy was started immediately after diagnosis. If necessary, the antibiotic therapy was changed after the antibiogram was available. X-ray control check with a water-soluble contrast agent was done after 8 h.

#### Type III

There is an esophageal perforation. Mediastinitis or pleural empyema may be present. In the case of a grade III perforation, suturing was performed, if necessary with a cervical sternocleidomastoid flap, an intercostal or latissimus flap depending on the anatomical localization. An internal and an external drainage with an endovacuum and two 24 Charriere chest tubes were placed. Pleural empyema was cleared if present. Antibiotic therapy was calculated immediately from the time of diagnosis, as soon as possible according to antibiogram.

#### Type IV

There is an older perforation with persistent fistula. Mediastinitis is also present. Sepsis, long-stretch rupture or esophageal necrosis may also be present. Esophagectomy with two-stage reconstruction was performed for grade IV perforation. Antibiotic therapy was calculated immediately, in the following according to antibiogram.

The classification takes into account the clinical presentation of the patient with signs of mediastinitis and sepsis, findings on CT scan and endoscopy. The patients can easily be categorized. The classification seems to establish a better stage-dependent comparability of patients, their treatment and outcome.

For further evaluation and as an outlook of research, it would be of interest to grade the known studies into the classification of esophageal perforation for a meta-analysis and conduct a prospective multicenter study in order to evaluate the feasibility and impact of this classification on survival and stage-dependent outcome in other centers. In case of the confirmation of the significant correlation between grading and survival, maybe even a differentiated therapy concept might be possible in the future.

## Data Availability

Raw datasets used can be accessed by MS.
